# Variability of clinical practice in the care of the second stage of labor among midwives in Spain

**DOI:** 10.1186/s12912-024-01863-7

**Published:** 2024-03-26

**Authors:** Estíbaliz Laderas Díaz, Julián Rodriguez-Almagro, Juan Miguel Martinez-Galiano, Rafael Picón Rodríguez, Antonio Hernández-Martínez

**Affiliations:** 1 Department of Obstetrics & Gynecology, La Mancha Centro General Hospital, Av. Constitución, 3, Alcázar de San Juan, Ciudad Real, 13600 Spain; 2 Department of Nursing, Physiotherapy and Occupational Therapy, Ciudad Real Faculty of Nursing, University of Castilla-La Mancha, Ciudad Real, Spain; 3 Department of Nursing, University of Jaen, Jaen, Consortium for Biomedical Research in Epidemiology and Public Health (CIBERESP), Madrid, Spain; 4 Department of General and Digestive Surgery, Santa B?rbara Hospital, Puertollano, Ciudad Real, 13500 Spain

**Keywords:** Variability, Midwife, Obstetrics, Professional practice, Second stage labor

## Abstract

**Background:**

There are recommendations based on scientific evidence on care in the second stage of labor, but it is not known to what degree the professionals comply with these recommendations.

**Objective:**

The aim of this study is to examine the variability in clinical practices among midwives during the second stage of labor, including positions, mobility, practices, and the maximum time allowed before initiating active pushing, and to assess their adherence to clinical practice guidelines.

**Methods:**

A cross-sectional observational study. A self-designed questionnaire was developed and distributed online through scientific societies. The main variables studied were professional and work environment characteristics, maternal positions and mobility, practices during this stage, maximum time to start active pushing and duration of the second stage of labor. Descriptive statistics were calculated using SPSS 24.0.

**Results:**

Regarding the woman’s position during childbirth, 80.3% (245) of midwives frequently or always allow the woman to choose her birthing position. Furthermore, 44.6% (136) of professionals prefer using side-lying positions for the mother. Regarding drinking fluids during childbirth, 51.1% (156) of midwives allowed the woman to drink the amount of liquids she wanted, whereas 11.1% (34) said that they would allow them to do so however, this was against the protocol of their hospital. When inquiring about the Kristeller maneuver, it was reported to be excessively performed in 35.1% (107) of cases for fetal bradycardia, 33.1% (101) for maternal exhaustion, and 38.4% (117) to avoid instrumental birth. Finally, a great variability was observed in the time criteria used for the initiation of active pushing and the maximum duration of the second stage of labor.

**Conclusions:**

Certain practices, such as the Kristeller maneuver, are overused among midwives, with great variability in the use of certain procedures, the waiting times to initiate pushing and completion of the second stage of labor. Further training and awareness campaigns are needed for professionals to apply evidence-based care.

**Supplementary Information:**

The online version contains supplementary material available at 10.1186/s12912-024-01863-7.

## Introduction

### Background

The second stage of labor or expulsion is the most critical stage of childbirth because of the risks involved for both the mother and the infant [1–3]. This stage requires close monitoring by the professionals attending the birth [4].

Regarding care in the second stage of labor, several studies have been published in recent years that have shown that certain interventions may be unnecessary and/or inappropriate [5–7]. In contrast, other interventions are scientifically supported to help to avoid or minimize certain complications, such as perineal trauma [8–13].

Based on this evidence, various scientific societies and agencies have developed clinical practice guidelines (CPG) and protocols to unify these recommendations. In 2018, The World Health Organization (WHO) published a series of recommendations on intrapartum care [14], as did the National Institute for Health and Care Excellence (NICE) [15]. Along these lines, in 2010, the Spanish Ministry of Health, Social Policy and Equality published the CPG on Normal Childbirth, which includes recommendations for the second stage of labor, such as: aseptic measures, maternal positions, the practice of pushing, measures for the prevention of perineal trauma, etc. [5]..

The use of these guidelines and documents is aimed at avoiding excessive variability in the clinical practice of childbirth care. This variability could lead to an increased risk of iatrogenesis due to inappropriate or unnecessary procedures, as well as not receiving care backed by scientific recommendations [16].

Despite this, adherence to these recommendations by professionals attending childbirth is currently unknown. Few studies have evaluated the variability in professional practice during this stage [16]. However, this information is highly relevant because of the implications for clinical practice and the possibility of reorienting professional education or strategies to improve adherence to evidence-based recommendations.

Therefore, this study is aimed to determine the variability of clinical practice among midwives during the second stage of labor.

## Methods

### Design and selection of study subjects

A cross-sectional observational study of midwives performing their healthcare activity in Spain in the year 2021. This study was approved by the Clinical Research Ethics Committee of Hospital Mancha-Centro with protocol number 194-C. The reference population used to estimate the sample size was 9593 active midwives, according to official statistics [17], a prevalence of the factor under study of 50% (this criterion was used because it was a multi-response questionnaire and the most conservative criterion), an absolute error of 6%, a replacement rate of 10% and a confidence level of 95%. This resulted in a minimum of 289 midwives under study.

### Sources of information

The questionnaire employed in this study was specifically designed by us, as there was no pre-existing instrument in the literature that covered the specific topics we aimed to explore. To construct this bespoke questionnaire, we convened a panel consisting of both the authors and external midwives with relevant expertise. This collaborative effort ensured the creation of a comprehensive set of questions and response options that accurately reflected our research objectives. Before its broader distribution, the questionnaire underwent a pilot phase. This preliminary testing was conducted with a select group of midwives from the Castilla-La Mancha association, allowing us to refine the questionnaire based on their feedback and ensure its clarity and effectiveness for capturing the intended data from a wider array of professional associations (Supplementary file 1).

The questionnaire consisted of 42 items (41 closed questions and one open question), three of which asked participants about sociodemographic data, nine focused on their activity and work environment, and the remaining 30 questions were about procedures used in the second stage of labor. The period of data collection was from October to December 2021. The type of sampling was a non-probabilistic convenience sample. The form was sent by email to the midwives via different professional associations and scientific societies of midwives, such as the Federation of Spanish Midwives Associations (FAME) and the National Association of Midwives of Spain, as well as regional associations. Those responsible for these associations individually sent the links to the questionnaire to guarantee that access was specifically for the population under study.

Before completing the form, the professionals had to read a sheet with information about the study, where they had to accept their consent to participate in it. Subsequently, information was provided on how to fill in the questionnaire, in addition, an e-mail was provided to resolve any possible doubts.

The variables collected in the study were as follows:

Work-related questions were included, such as the year of completion of training as a resident, the province in which the professional worked, the name of the work center (optional), whether the professional worked at a public center (yes/no), whether the professional worked at a private center (yes/no), whether the professional attended home births (yes/no/sometimes), the number of home births (yes/no/sometimes), if they worked in primary care (yes/no/sometimes), the number of births per year that took place at the hospital (< 500 births, 500–1000 births, 1001–2000 births, 2001–3000 births, 3001–4000 births, > 4000 births) and finally type of center according to the type of obstetric training provided (obstetricians and midwives) (If they did not train residents of midwives or obstetricians, if they only trained residents of midwives, if they only trained obstetricians or both specialties).

Several questions were asked in relation to the different techniques that the professional may or may not use during the second stage of labor, such as the intake of liquids, the use of oxytocin, maternal position, the performance of the Kristeller maneuver, the time elapsed for initiation of active pushing, the maximum time allowed according to parity and the use of epidural analgesia.

### Statistical analysis

A descriptive analysis was performed using absolute and relative frequencies for categorical variables and mean with standard deviation for quantitative variables. All analyses were performed using SPSS 24.0 statistical software.

### Ethical considerations

This study was approved by the Clinical Research Ethics Committee of Hospital Mancha-Centro with protocol number 194-C. Informed consent to participate was obtained from all participants in the study prior to their participation, indicating that they could withdraw at any time during the study. All work was performed in accordance with the principles of the Declaration of Helsinki (2013 version). All data were treated adhering to the General Data Protection Regulation (GDPR) 2016/679, 27th April 2016, and the Spanish Ley orgánica de protección de datos y garantía de derechos digitales (LOPDGDD; Data Protection And Guarantee Of Digital Rights) 3/2018, 5th December.

## Results

### Professional and work environment characteristics

A total of 305 midwives participated, of whom 90.5% (276) were women. Regarding the age of the professionals, 56.1% (171) were between 26 and 35 years old, 97% (296) worked in a public center, 56.8% (171) finished their residency training after 2015 and 11.5% (35) attended home births. Regarding their work center, 86.2% (263) worked in hospitals where residents were trained and 59.0% (180) worked in hospitals where between 1000 and 3000 births were attended per year. Table 1**(Sociodemographic characteristics and professional profile)** presents the professional and work environment characteristics in detail. Appendix 1. Distribution of participating midwives by province where they carry out their healthcare activity.

**Table 1:** Sociodemographic characteristics and professional profile

Variable	n (%) (*n* = 305)
**Age**	
≤ 25 years	19 (6.2)
26–35 years	171 (56.1)
36–45 years	71 (23.3)
46–55 years	35 (11.5)
56–65 years	9 (3.0)
**Sex**	
Male	29 (9.5)
Female	276 (90.5)
**Works at a public center**	
No	9 (3.0)
Yes	296 (97.0)
**Works at a private center**	
No	270 (88.5)
Yes	35 (11.5)
**Works in primary care**	
No	193 (63.3)
Yes	42 (13.8)
Sometimes	70 (23.0)
**Teaching hospital**	
No	42 (13.8)
Midwives only	16 (5.2)
Gynecologists only	7 (2.3)
Both specialties	240 (78.7)
**Hospital size**	
Up to 1000 births per year	69 (22.6)
1000 to 3000 births per year	180 (59.0)
Over 3,000 births per year	56 (18.4)
**Time since completion of midwifery training**	
Prior to 2005	36 (12.0)
2005–2015	94 (31.2)
2015–2021	171 (56.8)
Losses	4 (*n* = 301)
**Assists in home births**	
No	270 (88.5)
Yes	35 (11.5)

### Maternal mobility and positions during the second stage of labor

When asked about various practices during the second stage of labor, 80.3% (245) of midwives frequently or always let the woman choose the delivery position if the conditions were favorable, the position most used by the professionals when the woman had no preference was the side lying position 44.6% (136), the least used position was the delivery chair, chosen by 3.6% (11), whereas 84.3% (257) did not use the lithotomy position without foot support as the preferred option (**See** Table 2. **Questions on mobility and positions during the second stage of labor**).

**Table 2:** Questions on mobility and positions during the second stage of labor

Questions on mobility and positions	n (%) (*n* = 305)
**During the second stage of labor, if conditions are suitable, do you let the woman choose the birth position?**	
Never	2 (0.7)
Rarely	19 (6.2)
Occasionally	39 (12.8)
Frequently	104 (34.1)
Always	141 (46.2)
**If the pregnant woman has no preference for any position and the conditions permit it**	
**Do you prefer to use the standing position?**	
No	286 (93.8)
Yes	19 (6.2)
**Do you preferentially use the quadruped position?**	
No	274 (89.8)
Yes	31 (10.2)
**Do you preferentially use the birthing chair?**	
No	294 (96.4)
Yes	11 (3.6)
**Do you preferentially use side-lying positions?**	
No	169 (55.4)
Yes	136 (44.6)
**Do you preferentially use lithotomy without floor support?**	
No	257 (84.3)
Yes	48 (15.7)
**Do you preferentially use lithotomy with foot support?**	
No	189 (62.0)
Yes	116 (38.0)

### Practices during the second stage of labor

Regarding clinical practices during the second stage of labor, 76.1% (232) did not reduce the cervix manually during this stage, 49.5% (151) never used an antiseptic for vulvovaginal lavage prior to performing a vaginal examination. 61.6% (188) proposed spontaneous pushing in women without an epidural. Up to 51.1% (156) of the professionals allowed the woman to drink the amount of liquids she wanted and 11.1% (34) said that they would allow them to do so but that this was against the protocol at their hospital. In a normal delivery, 33.8% (103) would use oxytocin in the event of hypodynamia and if one hour of labor has elapsed. 71.1% (217) proposed spontaneous micturition before bladder catheterization only in women without an epidural. Regarding lidocaine spray to reduce perineal pain, it is never used by 81.3% (248). Lubricant is frequently used by 50.9% (155) to reduce the risk of tearing. When asked about the use of the Kristeller maneuver, the professionals responded that it was performed excessively in the case of fetal bradycardia 35.1% (107), in the case of maternal exhaustion 33.1% (101) and to avoid instrumental delivery 38.4% (117). After the end of labor, 37.4% (114) occasionally performed bladder catheterization **(**Table 3. **Questions on practices during the second stage of labor).**

**Table 3:** Questions on practices during the second stage of labor

Practices during the second stage of labor	n (%) (*n* = 305)
**During the second stage of labor, before performing a vaginal examination, do you use antiseptic for vulvovaginal lavage?**	
Never	151 (49.5)
Rarely	67 (22.0)
Occasionally	39 (12.8)
Frequently	30 (9.8)
Always	18 (5.9)
**Regarding pushing, in women without epidural anesthesia, do you propose spontaneous pushing?**	
Never	4 (1.3)
Rarely	5 (1.6)
Occasionally	13 (4.3)
Frequently	95 (31.1)
Always	188 (61.6)
**If the woman asks to drink fluids and the situation allows for it, what do you usually do?**	
I would let her drink, but my center’s protocol doesn’t allow me to do so	34 (11.1)
I wet her lips with a wet gauze	9 (3.0)
Yes, but only small amounts	106 (34.8)
I let her drink whatever she wants	156 (51.1)
**During the second stage, in a labor that has progressed normally, and the recording is normal, do you use oxytocin?**	
No	100 (32.8)
Only if there is hypodynamia (less than 3 contractions in 10 min) and one hour of expulsion has passed	103 (33.8)
Only if there is hypodynamia and 2 h have	69 (22.6)
Only if there is hypodynamia and 3 h have elapsed	26 (8.5)
Yes, I usually do it to shorten this phase	7 (2.3)
**During the second stage of labor, before performing a bladder catheterization, do you try to get the woman to void spontaneously?**	
No	26 (8.5)
Yes, but only when there is no epidural analgesia	217 (71.1)
Always	62 (20.3)
**Do you use local anesthetic such as lidocaine spray to reduce perineal pain at this stage?**	
Never	248 (81.3)
Rarely	25 (8.2)
Occasionally	19 (6.2)
Frequently	12 (3.9)
Always	1 (0.3)
**Do you use lubricant in the birth canal in order to reduce the risk of tearing?**	
Never	51 (16.7)
Rarely	45 (14.8)
Occasionally	54 (17.7)
Frequently	85 (27.9)
Always	70 (23.0)
**Is Kristeller performed at your center for fetal bradycardia?**	
It is never done	59 (19.3)
On very few occasions	139 (45.6)
It is performed excessively	107 (35.1)
**Is Kristeller performed at your center in the face of maternal exhaustion?**	
It is never done	84 (27.5)
On very few occasions	120 (39.3)
It is performed excessively	101 (33.1)
**Is Kristeller performed at your center to avoid instrumental delivery?**	
It is never done	70 (23.0)
On very few occasions	118 (38.7)
It is performed excessively	117 (38.4)
**After completing the second stage of labor, do you perform bladder catheterization?**	
Never	21 (6.9)
Rarely	73 (23.9)
Occasionally	114 (37.4)
Frequently	71 (23.3)
Always	26 (8.5)

### Time to initiate active pushing and to complete the second stage of labor

The next aspect evaluated was the time allowed to begin active pushing and to complete the second stage. In the first case, the most frequent responses were that in nulliparous women without an epidural, 44.6% (136) of the midwives would advise women to start active pushing after two hours of the second stage, in the case of nulliparous women with epidural 56.4% (172) would start pushing after two hours, in a multiparous woman without epidural 44.9% (137) would start pushing after one hour, in a multiparous woman with epidural 46.6% (142) would allow two hours to start pushing, and in a pregnant woman with a previous cesarean Sect. 40.3% (123) of midwives would allow two hours **(**Table 4. **Time allowed to initiate second stage pushes in second stage of labor).**

**Table 4:** Time allowed to initiate second stage pushes in second stage of labor

When a woman without pathology enters second stage of labor and there are no alterations in the fetal record and her condition is normal. How much time would you give until INITIAL ACTIVE PUSHING?							
Type of pregnant woman	Up to 1 h	Up to 1 h and 30 min	Up to 2 h	Up to 2 h and 30 min	Up to 3 h	Up to 3 h and 30 min	Unlimited
Nulliparous without epidural	55 (18.0)	43 (14.1)	**136 (44.6)**	24 (7.9)	19 (6.2)	3 (1.0)	25 (8.2)
Nulliparous with epidural	18 (5.9)	27 (8.9)	**172 (56.4)**	35 (11.5)	34 (11.1)	13 (4.3)	6 (2.0)
Multiparous without epidural	**137 (44.9)**	43 (14.1)	74 (24.3)	11 (3.6)	9 (3.0)	4 (1.3)	27 (8.9)
Multiparous with epidural	58 (19.0)	56 (18.4)	**142 (46.6)**	26 (8.5)	12 (3.9)	5 (1.6)	6 (2.0)
Pregnant with previous cesarean section	75 (24.6)	53 (17.4)	**123 (40.3)**	20 (6.6)	18 (5.9)	7 (2.3)	9 (3.0)

The most frequent response is presented in bold

Regarding the maximum time for completion of the second stage of labor, the most frequent response was that 36.4% (111) of the midwives would allow a nulliparous woman without an epidural three hours, in a nulliparous woman with epidural, 56.7% (173) of midwives would allow up to four hours, in a multiparous woman without epidural, 30.2% (92) would allow up to two hours, in a multiparous woman with epidural, 37.7% (115) would allow up to three hours and in a pregnant woman with previous cesarean section, 16.1% (49) would allow up to two hours, 22.3% (68) would allow to three hours, and 24.6% (75) would allow up to four hours **(**Table 5. **Maximum time allowed until the end of the second stage of labor).**

**Table 5:** Maximum time allowed until the end of the second stage of labor

When a woman with no pathology enters the second stage of labor and there are no alterations in the fetal registry and her condition is normal. What is the maximum time you would give to FINISH the second stage of labor?									
Type of pregnant woman	Up to 1 h	Up to 1 h and 30 min	Up to 2 h	Up to 2 h and 30 min	Up to 3 h	Up to 3 h and 30 min	Up to 4 h	Up to 5 h	Unlimited
Nulliparous without epidural	8 (2.6)	6 (2.0)	33 (10.8)	33 (10.8)	**111 (36.4)**	16 (5.2)	81 (26.6)	6 (2.0)	11 (3.6)
Nulliparous with epidural	6 (2.0)	4 (1.3)	12 (3.9)	36 (11.8)	36 (11.8)	16 (5.2)	**173 (56.7)**	18 (5.9)	4 (1.3)
Multiparous without epidural	22 (7.2)	9 (3.0)	**92 (30.2)**	46 (15.1)	60 (19.7)	17 (5.6)	44 (14.4)	4 (1.3)	11 (3.6)
Multiparous with epidural	10 (3.3)	5 (1.6)	37 (12.1)	38 (12.5)	**115 (37.7)**	23 (7.5)	67 (22.0)	8 (2.6)	2 (0.7)
Pregnant with previous cesarean section	17 (5.6)	30 (9.8)	49 (16.1)	35 (11.5)	68 (22.3)	20 (6.6)	**75 (24.6)**	5 (1.6)	6 (2.0)

The most frequent response is presented in bold

## Discussion

### Main findings

This study was carried out to determine the variability of the different childbirth practices during the second stage of labor, observing that most participating midwives let women choose the position of birth and if the woman does not have any preference, they use side lying positions or lithotomy with foot support. Regarding the use of the Kristeller maneuver, a significant proportion of midwives considered that it was used excessively at their work center. In the case of certain practices, such as the use of lubricant or the decision of whether to allow the woman to drink liquids during this process, there was a great variability in responses.

Furthermore, significant variability was also observed regarding the time allowed by professionals for initiation of active pushing during the second stage of labor and the time allowed to complete this period.

### Maternal positions during childbirth

In relation to birthing positions used by mothers, there are publications that have observed that the squatting or chair position reduces pain in the second stage of labor [18], and that perineal trauma is reduced in side lying positions [19]. In a systematic review comparing the supine versus side lying position, it was also observed that the latter was associated with a significant reduction in the duration of labor [20].

In the same vein, a systematic review published in 2017 concluded that the upright position compared to supine positions, was associated with a reduction in the duration of the second stage of labor, as well as a decrease in instrumental births and episiotomies, although a possible increase in second-degree perineal tears was observed (20–22). Despite this, some authors believe that women should not be discouraged from adopting these positions to prevent perineal damage [21] and women should be encouraged to use the position that is most comfortable for her [22].

Nonetheless, no literature has been found to report which position is most frequently used in clinical practice during the third stage of labor. In April 2022, a study describing midwives’ perspectives on culturally appropriate care to support maternal positions highlighted the importance of including women in decision-making regarding their birth preferences [23]. In our study, most of the practitioners offered to allow the woman to assume the position of her choice, and if the woman had no preference, most opted for side lying positions or lithotomy with foot support.

### Maternal active pushing

Regarding pushing, the WHO considers that sustained (Valsalva maneuver) and directed pushes are not effective and are also harmful and therefore not a recommended practice [5]. In our study, midwives agreed with these recommendations and when asked about pushing in women without epidurals, most midwives suggested spontaneous pushing. Moreover, in a meta-analysis of randomized trials (21 trials, 3763 participants) comparing different pushing techniques, it was concluded that maternal and neonatal outcomes were similar in women who followed their own pushing instincts (spontaneous pushing) and those who performed directed pushing [24].

### Fluid intake, bladder catheterization, and analgesia

In this regard, restrictive policies are still very broad, perhaps because they depend on several professionals (anesthesiologists, gynecologists and midwives), although the WHO recommends fluid intake during labor in low-risk women [14]. Another prospective study with 249 cases supported this recommendation [25]. Despite these recommendations, a significant number of midwives who responded to our study reported that they would be open to give fluids to the pregnant woman however this was against their hospital protocol, and of those who did, many reported that they only give liquids in small amounts.

In the same line, the SEGO (Sociedad Española de Ginecología y Obstetrícia) also recommend encouraging spontaneous urination, and if this is not possible or the amount is insufficient, bladder catheterization should be performed [26]. According to our data, most midwives would do this in a woman without an epidural but not if the mother had epidural analgesia.

Regarding the use of local anesthetic spray, we found only one study with 185 women that concluded that the use of local anesthetic spray is not associated with a decrease in pain during birth, although it may be associated with a reduction in perineal trauma [27]. In our study most midwives do not use it as a means of pain relief at this stage.

### Kristeller maneuver

Concerning the Kristeller maneuver, there are currently no records in the mother’s clinical history on its use, which makes it difficult to collect data on its incidence. In 2008, the SEGO stated that it should only be used in very specific cases and never to assist in the descent of head planes [26]. The WHO guide for the care of normal childbirth states that it is a practice that entails risks for both the mother and the infant and although there are not many studies on the subject, the general impression is that it is used excessively [5]. The results of our study agree with this statement, since a large number of professionals consider that it is used excessively in their workplace.

### Duration of the second stage of labor

There is no standardized definition of duration of labor, nor is there consensus on which guideline is most appropriate for clinical use [28]. The Ministry of Health and Social Policy in 2010 established that the normal duration of the passive phase in nulliparous women is up to two hours whether or not they have epidural anesthesia. The passive phase in multiparous women is up to one hour if they do not have epidural anesthesia and up to two hours if they do. Regarding the duration of the active phase, in nulliparous women it is up to one hour if they do not have epidural anesthesia and up to two hours if they do. In multiparous women it is up to one hour whether or not they have epidural anesthesia [5]. In our study we observed variability regarding the times granted by each professional, this variability is especially marked when the pregnant woman has had a previous cesarean section. This situation of a pregnant woman with a previous cesarean section who attempts vaginal birth is not contemplated in many clinical practice guidelines [29, 30]. These mentioned that this stage should not exceed two hours in nulliparous women and 1.5 h in multiparous women; it does not refer to whether or not they are under epidural analgesia. The Spanish protocol of the Hospital Clinic of Barcelona “Gestational control in pregnant women with previous cesarean section”, states that the expulsion should not last more than three hours [31]. Other authors point out that the duration in these pregnant women should be the same as in women without previous cesarean section although more caution should be exercised and with an even closer follow-up [32]. Finally, a retrospective cohort study involving 198 women concluded that the different practices used by professionals attending the birth influenced the duration of the same [33].

### Strengths and limitations

Among the limitations of this study, there is a risk of selection bias, since it is possible that the midwives who participated were more sensitive to the study subject. This could mean that the true results present even greater variability compared to our findings. Moreover, we have also observed that the age of the participants is relatively young, which could give us an insight into the future regarding the trend in the evolution of care during the second stage of labor. However, determining the response rate is challenging due to varying levels of engagement from associations in distributing the questionnaire to their members.

Among the strengths of this study, it is worth highlighting that it is the first study in Spain to evaluate the variability in the clinical practice of midwives during the second stage of labor. We have only found one study in the United Kingdom which sought to research the practices of midwives at the time of birth, which concluded that they additional training was needed on the identification of anal sphincter lesions [34]. The results of this study could serve as a basis for further research, as well as comparative research aimed at raising awareness of the need for adherence to evidence-based recommendations in clinical practice.

### Implications for clinical practice and research

A recent study by Soriano-Vidal et al. [35] in Spain highlights certain evidence-based practices—such as support during childbirth, permitting fluid intake, and skin-to-skin contact—that have been shown to significantly enhance maternal satisfaction. Reducing practice variability among midwives by adhering to clinical practice guidelines is crucial for both improving patient safety and enhancing perceptions of care received. However, there has been no data collection to date on professionals’ compliance with these guidelines. This gap underlines the importance of our work in providing insight into current childbirth care practices, identifying areas with the most significant deficiencies, and designing policies for professional training.

## Conclusions

We can affirm that in Spain there is an important variability in the practices of midwives regarding management of the second stage of labor, at times because they do not conform to the recommendations, furthermore, there does not seem to be a clear consensus on certain practices.

It is necessary to increase efforts in the training and awareness of professionals to try to reduce this variability in clinical practice in line with scientific recommendations.

### Electronic supplementary material

Below is the link to the electronic supplementary material.

Supplementary Material 1

Supplementary Material 2

## Data Availability

The datasets produced during the current study are available from the corresponding author upon reasonable request.
