# Supplementary material for: Variability of clinical practice in the care of the second stage of labor among midwives in Spain
Source: BMC Nurs. 2024 Mar 26;23:202. doi: 10.1186/s12912-024-01863-7 (PMC10964659; doi:10.1186/s12912-024-01863-7)
Supplement: Supplementary file 1 — Supplementary Material 1 [file 12912_2024_1863_MOESM1_ESM.docx]

**Appendix 1. Distribution of participating midwives by province where they carry out their healthcare activity**

| **Province** | | **Frequency** | **Percent** |
| --- | --- | --- | --- |
| Valid | Álava | 2 | 0.7 |
|  | Albacete | 4 | 1.3 |
|  | Alicante | 12 | 3.9 |
|  | Almería | 6 | 2.0 |
|  | Asturias | 4 | 1.3 |
|  | Ávila | 2 | 0.7 |
|  | Badajoz | 5 | 1.6 |
|  | Barcelona | 11 | 3.6 |
|  | Burgos | 1 | 0.3 |
|  | Cáceres | 1 | 0.3 |
|  | Cádiz | 18 | 5.9 |
|  | Cantabria | 3 | 1.0 |
|  | Castellón | 3 | 1.0 |
|  | Ciudad Real | 29 | 9.5 |
|  | Córdoba | 5 | 1.6 |
|  | Girona | 2 | 0.7 |
|  | Granada | 7 | 2.3 |
|  | Guadalajara | 2 | 0.7 |
|  | Guipúzcoa | 7 | 2.3 |
|  | Huelva | 3 | 1.0 |
|  | Islas Baleares | 10 | 3.3 |
|  | Jaén | 21 | 6.9 |
|  | La Coruña | 4 | 1.3 |
|  | La Rioja | 1 | 0.3 |
|  | Las Palmas | 11 | 3.6 |
|  | León | 1 | 0.3 |
|  | Lleida | 2 | 0.7 |
|  | Madrid | 43 | 14.1 |
|  | Málaga | 7 | 2.3 |
|  | Murcia | 10 | 3.3 |
|  | Navarra | 4 | 1.3 |
|  | Orense | 1 | 0.3 |
|  | Palencia | 1 | 0.3 |
|  | Pontevedra | 6 | 2.0 |
|  | Salamanca | 2 | 0.7 |
|  | Santa Cruz de Tenerife | 17 | 5.6 |
|  | Sevilla | 5 | 1.6 |
|  | Tarragona | 1 | 0.3 |
|  | Teruel | 1 | 0.3 |
|  | Toledo | 4 | 1.3 |
|  | Valencia | 7 | 2.3 |
|  | Valladolid | 4 | 1.3 |
|  | Vizcaya | 5 | 1.6 |
|  | Zamora | 1 | 0.3 |
|  | Zaragoza | 9 | 3.0 |
|  | Total | 305 | 100.0 |
