# Supplementary material for: Variability of clinical practice in the care of the second stage of labor among midwives in Spain
Source: BMC Nurs. 2024 Mar 26;23:202. doi: 10.1186/s12912-024-01863-7 (PMC10964659; doi:10.1186/s12912-024-01863-7)
Supplement: Supplementary file 2 — Supplementary Material 2 [file 12912_2024_1863_MOESM2_ESM.docx]

QUESTIONNAIRE ON PROCEDURES USED DURING THE SECOND STAGE OF LABOR

Dear obstetrics professional, we, a group of maternal and child health professionals, invite you to participate in a research study titled “Variability in Professional Practice during the Second Stage of Labor”.

This study aims to understand the practices commonly performed by obstetrics professionals in Spain during the second stage of labor, or the expulsion phase.

This questionnaire is completely anonymous and voluntary. It will only take about 10 minutes of your time, but the information you provide could be of great value in understanding the current situation.

If you have any questions or suggestions, please contact the lead researcher: Antonio Hernández Martínez, Ph.D. in Socio-health Research and Professor at the Faculty of Nursing in Ciudad Real, via the following email address: xxxxxxx

1. Do you agree to participate in the study?
   - No
   - Yes
2. Do you have any conflict of interest with this study?
   - No
   - Yes
3. How old are you?
4. Gender
   - Male
   - Female
5. Year of residency completion
6. In which province do you carry out your professional activity?
   - Alava
   - Albacete
   - Alicante
   - Almería
   - Asturias
   - Avila
   - Badajoz
   - Barcelona
   - Burgos
   - Caceres
   - Cadiz
   - Cantabria
   - Castellon
   - Ciudad Real
   - Cordoba
   - Coruña, A
   - Cuenca
   - Girona
   - Granada
   - Guadalajara
   - Guipuzcoa
   - Huelva
   - Huesca
   - Illes Balears
   - Jaen
   - Leon
   - Lleida
   - Lugo
   - Madrid
   - Malaga
   - Murcia
   - Navarra
   - Ourense
   - Palencia
   - Las Palmas
   - Pontevedra
   - La Rioja
   - Salamanca
   - Segovia
   - Sevilla
   - Soria
   - Tarragona
   - Santa Cruz de Tenerife
   - Teruel
   - Toledo
   - Valencia
   - Valladolid
   - Vizcaya
   - Zamora
   - Zaragoza
7. Please indicate the name of your workplace if it is not an inconvenience (optional)
8. Do you work in a public center?
   - No
   - Yes
9. Do you work in a private center?
   - No
   - Yes
10. Do you assist in home births?
    - No
    - Yes
11. Do you work in primary care?
    - No
    - Yes
12. How many births are performed at your hospital annually? If you work in two centers, the answer should refer to the larger center.
    - <500 annual births
    - 500-1000 annual births
    - 1000-2000 annual births
    - 2000-3000 annual births
    - 3000-4000 annual births
    - 4000 annual births
13. Are residents trained at your hospital?
    - No
    - Yes
14. In which specialties?
    - Midwifery only
    - Gynecology only
    - Both specialties

ON CLINICAL PRACTICE DURING THE SECOND STAGE OF LABOR

1. During the second stage of labor, if conditions allow, do you let the woman choose the birthing position?
   - Never
   - Rarely
   - Occasionally
   - Frequently
   - Always

If the pregnant woman has no preference for any position and conditions allow

16. Do you preferentially use standing position? - No - Yes

1. Do you preferentially use all-fours position?
   - No
   - Yes
2. Do you preferentially use the birthing chair?
   - No
   - Yes
3. Do you preferentially use side-lying positions?
   - No
   - Yes
4. Do you preferentially use lithotomy position without plantar support?
   - No
   - Yes
5. Do you preferentially use lithotomy position with plantar support?
   - No
   - Yes
6. During the second stage of labor, before performing a vaginal examination, do you use antiseptic for vulvovaginal washing?
   - Never
   - Rarely
   - Occasionally
   - Frequently
   - Always
7. Regarding pushing, in women without epidural anesthesia, do you propose spontaneous pushing?
   - Never
   - Rarely
   - Occasionally
   - Frequently
   - Always
8. If the woman asks to drink fluids and the situation allows, what do you usually do?
   - I would let her drink, but my center's protocol does not allow it.
   - I wet her lips with a damp gauze.
   - Yes, but only small amounts.
   - I let her drink whatever she wants.
9. During the second stage, in a labor that has progressed normally and the monitoring is normal, do you use oxytocin?
   - No
   - Only if there is hypodynamia (less than 3 contractions in 10 minutes) and it has been an hour of expulsion.
   - Only if there is hypodynamia (less than 3 contractions in 10 minutes) and it has been two hours of expulsion.
   - Only if there is hypodynamia (less than 3 contractions in 10 minutes) and it has been three hours of expulsion.
   - Yes, I usually do it to shorten this phase.
10. During the second stage of labor, before performing a bladder catheterization, do you try to make the woman perform a spontaneous urination?
    - Always
    - Yes, but only when she does not have epidural analgesia
    - No
11. Do you use local anesthetic such as lidocaine spray to reduce perineal pain at this stage?
    - Never
    - Rarely
    - Occasionally
    - Frequently
    - Always
12. Do you use lubricant in the birth canal with the aim of reducing the risk of tearing?
    - Never
    - Rarely
    - Occasionally
    - Frequently
    - Always
13. Is Kristeller maneuver performed in your center in the case of fetal bradycardia?
    - It is never done
    - On very few occasions
    - It is performed excessively
14. Is Kristeller maneuver performed in your center in the case of maternal exhaustion?
    - It is never done
    - On very few occasions
    - It is performed excessively
15. Is Kristeller maneuver performed in your center to avoid instrumental delivery?
    - It is never done
    - On very few occasions
    - It is performed excessively
16. After completing the second stage of labor, do you perform bladder catheterization?
    - Never
    - Rarely
    - Occasionally
    - Frequently
    - Always

When a woman without pathology enters the second stage of labor and there are no alterations in fetal monitoring and her condition is normal, how long would you wait until STARTING ACTIVE PUSHING?

1. Nulliparous without epidural
   - Up to 1 hour
   - Up to 1 hour and 30 minutes
   - Up to 2 hours
   - Up to 2 hours and 30 minutes
   - Up to 3 hours
   - Up to 3 hours and 30 minutes
   - No limits
2. Nulliparous with epidural
   - Up to 1 hour
   - Up to 1 hour and 30 minutes
   - Up to 2 hours
   - Up to 2 hours and 30 minutes
   - Up to 3 hours
   - Up to 3 hours and 30 minutes
   - No limits
3. Multiparous without epidural
   - Up to 1 hour
   - Up to 1 hour and 30 minutes
   - Up to 2 hours
   - Up to 2 hours and 30 minutes
   - Up to 3 hours
   - Up to 3 hours and 30 minutes
   - No limits
4. Multiparous with epidural
   - Up to 1 hour
   - Up to 1 hour and 30 minutes
   - Up to 2 hours
   - Up to 2 hours and 30 minutes
   - Up to 3 hours
   - Up to 3 hours and 30 minutes
   - No limits
5. Pregnant woman with previous cesarean
   - Up to 1 hour
   - Up to 1 hour and 30 minutes
   - Up to 2 hours
   - Up to 2 hours and 30 minutes
   - Up to 3 hours
   - Up to 3 hours and 30 minutes
   - No limits

When a woman without pathology enters the second stage of labor and there are no alterations in fetal monitoring and her condition is normal, what is the MAXIMUM time you would allow to COMPLETE the second stage of labor?

1. Nulliparous without epidural
   - Up to 1 hour
   - Up to 1 hour and 30 minutes
   - Up to 2 hours
   - Up to 2 hours and 30 minutes
   - Up to 3 hours
   - Up to 3 hours and 30 minutes
   - Up to 4 hours
   - Up to 5 hours
   - No limits
2. Nulliparous with epidural
   - Up to 1 hour
   - Up to 1 hour and 30 minutes
   - Up to 2 hours
   - Up to 2 hours and 30 minutes
   - Up to 3 hours
   - Up to 3 hours and 30 minutes
   - Up to 4 hours
   - Up to 5 hours
   - No limits
3. Multiparous without epidural
   - Up to 1 hour
   - Up to 1 hour and 30 minutes
   - Up to 2 hours
   - Up to 2 hours and 30 minutes
   - Up to 3 hours
   - Up to 3 hours and 30 minutes
   - Up to 4 hours
   - Up to 5 hours
   - No limits
4. Multiparous with epidural
   - Up to 1 hour
   - Up to 1 hour and 30 minutes
   - Up to 2 hours
   - Up to 2 hours and 30 minutes
   - Up to 3 hours
   - Up to 3 hours and 30 minutes
   - Up to 4 hours
   - Up to 5 hours
   - No limits
5. Pregnant woman with previous cesarean
   - Up to 1 hour
   - Up to 1 hour and 30 minutes
   - Up to 2 hours
   - Up to 2 hours and 30 minutes
   - Up to 3 hours
   - Up to 3 hours and 30 minutes
   - Up to 4 hours
   - Up to 5 hours
   - No limits
